# Natural language processing analysis of the psychosocial stressors of mental health disorders during the pandemic

**DOI:** 10.1038/s44184-023-00039-6

**Published:** 2023-10-05

**Authors:** María P. Raveau, Julián I. Goñi, José F. Rodríguez, Isidora Paiva-Mack, Fernanda Barriga, María P. Hermosilla, Claudio Fuentes-Bravo, Susana Eyheramendy

**Affiliations:** 1 Faro, Universidad del Desarrollo, Santiago, Chile; 2 DILAB, Facultad de Ingeniería, Pontificia Universidad Católica de Chile, Santiago, Chile; 3 Science, Technology, and Innovation Studies, The University of Edinburgh, Edinburgh, Scotland; 4 Facultad de Ingeniería y Ciencias, Universidad Adolfo Ibáñez, Santiago, Chile; 5 Escuela de Psicología, Universidad Adolfo Ibáñez, Santiago, Chile; 6 GobLab, Escuela de Gobierno, Universidad Adolfo Ibáñez, Santiago, Chile; 7 Fundación Todo Mejora, Santiago, Chile; 8 Facultad de Derecho, Universidad de Chile, Santiago, Chile

**Keywords:** Human behaviour, Decision making

## Abstract

Over the past few years, the COVID-19 pandemic has exerted various impacts on the world, notably concerning mental health. Nevertheless, the precise influence of psychosocial stressors on this mental health crisis remains largely unexplored. In this study, we employ natural language processing to examine chat text from a mental health helpline. The data was obtained from a chat helpline called *Safe Hour* from the “It Gets Better” project in Chile. This dataset encompass 10,986 conversations between trained professional volunteers from the foundation and platform users from 2018 to 2020. Our analysis shows a significant increase in conversations covering issues of self-image and interpersonal relations, as well as a decrease in performance themes. Also, we observe that conversations involving themes like self-image and emotional crisis played a role in explaining both suicidal behavior and depressive symptoms. However, anxious symptoms can only be explained by emotional crisis themes. These findings shed light on the intricate connections between psychosocial stressors and various mental health aspects in the context of the COVID-19 pandemic.

## Introduction

Mental health may become the next pandemic^1^. Recent studies show that the global prevalence of depression has risen from 9.6% to 28% and anxiety from 12.9% to about 26%^2,3^. During the COVID-19 crisis, about 16.4% of the global population experienced suicidal thoughts, and over 50% of the population showed symptoms of loneliness, stress, and low levels of well-being^2^. These studies link mental health issues to structural inequality, poverty, and countries’ response capabilities^2,3^. In a post-pandemic world, governments will have to deal with the mental health consequences in a context of continuing distress produced by the likely economic recession^4^. Moreover, this scenario has shown the need to rethink and drastically improve public health services for the future^5,6^.

Assessing the mental health impact of the COVID-19 pandemic is challenging as it requires information and data gathered before and during the pandemic^7^. Many countries have missed out on the opportunity to assess the mental health impact of COVID-19, as they did not have a baseline or control data to compare new information^7^. Most mental health population studies depend on large-scale self reporting^8–11^. However, conducting such large-scale population studies can be costly^12,13^. This has led to a paradigm shift in many fields of research. For instance, human-mobility studies used to rely on the active solicitation of data through travel surveys and self-reports, but has since embraced inferences based on computational analysis of passive data generated by cell phones users^14^. Analyzing direct behavior can also lead to more precise interpretations. For instance, researchers have found that liberals tend to self-report less happiness than conservatives, but display more in their actual behavior^15^.

More recently, computer-based tools, such as Natural Language Processing (NLP) and Machine Learning (ML), have increasingly been adopted to study mental health^16^. Using large amounts of text from either patient records, emergency room data or even social media, researchers have been able to extract symptoms, classify the severity and identify psycho-pathological indicators^16^. NLP has even been used to design chatbots for complementary mental health treatment^17^. Combining linguistics and computer science, researchers have tested automated markers for mental health, such as excessive self-focus shown by first-person pronouns and negative emotions using word dictionaries^18^. Recent studies have sought to use these new computational approaches for population studies in mental health using non-clinical data^19^. They often rely on social media data and the Linguistic Inquiry and Word Count (LIWC) dictionary^20,21^. Other studies also use machine learning/deep learning approaches to inductively assess mental health symptoms in social media forums and communities^12,22^.

Dictionary approaches for characterizing mental health problems entail formulating word groups associated with specific psychological constructs, followed by analysing the text frequencies of these words^23^. Such methods inherently presuppose the existence of a data ontology or taxonomy that connects terms in conceptually meaningful ways^23^. For instance, the LIWC proposes a series of words that are hypothesized to relate to particular emotions, cognitive processes and social relations and does not need a model to make any inference. On the other hand, deep learning approaches involve supervised training of algorithms using neural networks to estimate the model for classification. Nonetheless, there are some serious limitations of the current uses of both approaches that should be considered.

"Off-the-Shelf” dictionaries^24^ such as the LIWC provide stable and rich markers for psychological constructs and have now been translated into multiple languages. However, these dictionaries are context-blind^25^ because they do not account for meaning changes in words depending on the full context of the phrase and its use in different “language games”^26^. General use dictionaries, such as the LIWC, have top-down bias, as they operate with pre-defined ontologies that are assumed to be stable across domains and discourses, which can lead to significant inaccuracies^24^. Most population-level studies using dictionaries utilize rough sentiment analysis to measure the mood valence and emotion shifts over time^19^. This is because current markers of the LIWC can only serve as features and complementary data in more specific mental health studies. Despite the value of its “emotion”, “cognitive processes” and “social relation markers”, the LIWC lacks more specific mental health constructs, such as symptoms or psychopathology markers. For this reason, computational studies still rely on self-reports to assess mental health symptoms and other domain-specific constructs^21^.

Machine learning and, particularly, deep learning studies have shown great accuracy in predicting the mental health status of people using their social media data^27–29^. However, Deep Learning models are perceived as “black boxes" in which inputs are computed and conclusions are reached without too much explanation of their inner workings^27^. This lack of transparency is critical when trying to convince mental health experts to embrace the possibilities and outcomes of machine learning models^27^. Moreover, understanding why an algorithm is making certain “decisions” is relevant for learning about that phenomenon. There have been discussions about incorporating Explainable Artificial Intelligence (XAI) techniques for making sense of algorithmic decision-making in health science, but this is still a pending challenge^30^. Some studies include mental health experts, but mostly for labeling data and not for co-constructing the conceptual underpinnings used for interpretations of the data^31^.

Social media has proven to be an effective source of big data for mental health analysis^31^. However, the informality of social media data and its public availability raises questions about its quality and ability to protect the privacy and anonymity of participants^12,16^. This makes it a sub-optimal data source alternative in comparison to clinical interviews and notes or other forms of clinical data, in which practitioners can exercise content regulation^31^. Although presumably of higher quality, these records would likely be difficult to acquire in the necessary volume because of institutional restrictions in the public sector, or the lack of a centralized source of data in the private sector.

As an alternative, mental health helplines are a growing and global phenomenon. Just in the UK there are over 2500 helplines in operation^32^.The International Federation of Telephone Emergency Services (IFOTES) estimates that four million telephone conversations are held every year in Europe^33^. In these helplines (chat-based, telephone-based and mixed), conversations are held between participants and paid workers or trained volunteers. Because of this, discussions deliberately cover important information pieces to some degree consistently across users, which is necessary for better data interpretations^31^. In sum, these data sources seem to provide higher quality data than social media, with rich free-text and higher accessibility than medical files and interviews.

Because of its conversational nature, data from these mental health helplines may also help researchers explore a population’s mental health beyond the prevalence of symptomatology. Recent studies have sought to explore the connections between specific symptoms, such as depression or anxiety and potential intermediate variables during and beyond the pandemic, such as demographics and occupational data^34^, stressful life events^35^, financial conditions^36,37^, and isolation due to confinement^38^, among others. These intermediate variables cast a light on the psychosocial stressors behind mental health symptoms often absent from large-scale mental health studies. Psychosocial stressors are key for tackling a population’s mental health, as they can hold an explanatory value for symptoms^39^. However, because of the dialogical nature of helpline conversations, there is a challenge for data analysis in identifying and controlling by volunteer strategies that may elicit certain user responses.

In sum, there is an opportunity to utilize NLP approaches to large-scale mental health research beyond the use of social media data. We assert that helplines could provide a higher-quality alternative and open up new venues for computational analysis. In this work, we study helpline chat data from the *Safe Hour* program of *Fundación Todo Mejora* (the “It Gets Better” project) in Chile. We aim to assess the effect of the pandemic on psychosocial stressors and the relationship between stressors and three mental health issues: suicidal behavior, anxious symptomatology, and depressive symptomatology, in order to address the following research questions: (1) What are the main psychosocial stressors present in helpline conversations?, (2) How did the relative prevalence of psychosocial stressors change before and after the pandemic?, (3) How do psychosocial stressors relate to mental health issues?.

## Methods

### Expert interviews and initial tagging

We first selected seven volunteers from the Foundation for interviews. From these interviews, we inductively identified psychosocial stressors by proxy of the themes of conversations in the helpline. We also identified volunteer strategies to use them as control variables. Once we had identified the categories in which each conversation can be classified, we selected six expert volunteers from the foundation plus two professional psychologists from our team, in order to manually label a set of 1000 conversations to train our models. The tagging process included conversations themes, volunteer strategies and the three mental health issues (suicidal behavior, anxious and depressive symptomatologies).

### Raw data

This study uses the data of a Chilean-based NGO helpline that provides free-access mental health support through chat. Overall, our sample consisted of 45,944 conversations, of which 10,986 were included in this article after removing conversations with less than 10 messages. Of these, 2335 were gathered in 2018, 4974 in 2019, and 3701 in 2020. Participants were able to freely decide to share personal information when reaching out to the helpline. During these three years, 4643 of 7640 participants revealed their age. Out of these participants, the average age was 18.89 years old (5.17 years Standard Deviation). The average amount of words per conversation in our final database was 742. The total amount of different volunteers registered in the NGO was 210 from 2018 to 2020.

Since the conversations in the database included personal and sensitive data, we carried out a data protection impact assessment, led by a data protection expert. This helped the research team identify risks and take measures to eliminate or mitigate them. The data protection measures included a data transfer agreement between the university and the “It Gets Better” project in Chile with General Data Protection Regulation (GDPR) standards. All team members signed confidentiality agreements with the university. The data was transferred by a secure method. The research team and Foundation members also received specialized training on data protection at the beginning of the project. The data was obtained by the “It Gets Better” project in Chile through its “Safe Hour” program. The consent of the users of that program includes the use of the data for research. The ethical approval was obtained from the Comité Ético de Investigación, Universidad Adolfo Ibáñez, No. 02/2021. Finally, the lead data scientist anonymized the data before the analysis.

### Identification of conversational themes and volunteers’ intervention types

In order to identify the key dimensions that structure mental health conversations within our sample, we conducted a qualitative investigation. For this qualitative study, we undertook semi-structured in-depth interviews^40^ with expert volunteers of the NGO. In this sense, we utilized purposive sampling through a critical case approach^41^. Supervisor recommendations alongside user evaluations were used to determine the expertise of volunteers. The inclusion criteria were set as belonging to the top quartile in the user assessment and were recommended by a supervisor.

The topics covered by the in-depth interviews included the following:

- Representations of “a typical conversation”: Questions related to the development of a typical case conversation.
- Perceived typology of users and cases: Questions related to volunteer perceptions about what type of users approach the helpline and what type of issues they present.
- Expert response patterns associated with different case scenarios: Questions about volunteer responses according to the different type of user cases.

We conducted seven in-depth interviews, each lasting approximately one and a half hours. The resulting audio files were professionally transcribed and analyzed using thematic analysis^42^ to detect patterns through reading, coding, and discussing transcripts of qualitative information. We used an investigator triangulation approach^43^ in which a subset of researchers held critical discussions about coding and the thematic structure of the text. After the fifth interview, qualitative saturation^44^ was achieved using the criteria of code saturation^45^, as no new themes emerged from the data. This reflects on the highly consistent set of practices that conform to the expert knowledge of these volunteers. The last interviews were used to add nuances to existing schemes and to confirm saturation. Analytic memos^46^ were iteratively written throughout the qualitative research process in order to raise emergent categories. Finally, we developed a list of thematic families and volunteer interventions that served as the data ontology of these conversations. The final categories were triangulated^43^ with direct analysis of a random sample of 20 conversations. The selection criteria for this random sample were having more than 10 messages, and balancing conversations that the NGO tagged as containing depressive or anxious symptomatology and/or suicidal behavior.

### Labeling of conversations

A significant portion of the 10,986 conversations was either inadequately tagged or untagged. Therefore, an expert tagging process was employed to construct and validate data analysis tools. The goal of the process was to label 1000 random conversations. At first, two mental health experts of the research team independently tagged a random sample of 200 conversations (with more than 10 messages), based on the qualitative categories (thematic families and volunteer interventions) collected in the interviews. Additionally, they tagged the conversations for the three main mental health issues that concern the NGO, namely, depressive and anxious symptomatologies, and suicidal behavior. We used these issues as the basis for the tagger’s comparison because they implicate the highest interpretative load and thus represent the most conservative standard. Of the 200 conversations, agreement was achieved at a rate of 85% in depressive symptomatology, 88% in anxious symptomatology, and 89% in suicidal behavior.

By labeling the first 200 conversations, our experts were able to improve the set of categories that had been previously inferred from the interviews. In addition, they gathered enough experience to train volunteers to tag the largest part of the conversations. Thus, NGO volunteers were invited to tag a greater volume of conversations. They were selected based on a test in which they tagged 10 random cases previously marked by our two experts in complete agreement. Volunteers were required to correctly tag at least 80% of conversations according to each displayed symptomatology. Overall, 12 volunteers took the test and 6 passed. The conversations tagged by the 6 volunteers, plus the 200 conversations tagged by our experts, added up to 1000 conversations.

To assess how the relative frequency of thematic families changed during the COVID-19 pandemic, we built a dictionary of thematic families. In this context, a dictionary is a structure of words and categories, where each word is associated with one or more categories. In our dictionary, each category corresponds to one thematic family. The set of words belonging to each category was derived as follows:

- Using the sample of 1000 conversations tagged by the experts, we trained a Random Forest classifier for each thematic family. We set up a Tf-idf matrix with tokens and bigrams to train the classifier. Preprocessing included lowercasing, stop-word removal, lemmatizing and noun-adjectives-verb filtering. For all of these processes, we used different libraries in Python, such as *sklearn* for the classifier, *nltk* and *stanza* for text-mining, and natural language processing.
- We then selected the 40 most predictive features in each case and generated a preliminary list of words.
- Then, if any word in a conversation was contained by the dictionary, the conversation was labeled according to the corresponding category.
- We evaluated the dictionary’s performance by comparing its classification with that of the experts. In this phase, some words were added or removed to improve the dictionary’s performance.
- The lists of words were refined by our psychology experts.

### Model-based assessment of thematic families frequency change pre/post pandemic

Once the dictionary was ready, we applied it to the entire conversation dataset. We then counted how many conversations were labeled in each thematic family by month and year, normalized by the total number of conversations. Following this, we compared the relative presence of the thematic family before and after the pandemic reached Chile (March 2020), by comparing the prevalence between both periods. This comparison was performed by setting up a first-order autoregressive model on thematic prevalence and adding a dummy variable to indicate the pandemic months. Other covariates, such as city lockdowns, mask policy, social-distancing policy or vaccination were not included. Furthermore, our dataset did not systematically include geographical references. Given that Chile adopted a dynamic COVID-19 policy in which events such as lockdowns and other restrictions changed periodically and locally, according to level of contagion, without that critical information we were not be able to determine those specific events at any given time.

### Logistic regressions to identify the conversational themes associated with mental health issues

To assess how the thematic families were related to different mental health issues, we set up a logistic regression model for each one: suicidal behavior, depressive and anxious symptomatology. We used the 1000 conversation dataset tagged by the experts to train these models. The independent variables were the presence/absence of the six thematic families and seven forms of interventions identified in each conversation by the experts. Thus, all regressors were binary variables, and the reference category was 0 (absence).

### Reporting summary

Further information on research design is available in the Nature Research Reporting Summary linked to this article.

## Results

### Identification of thematic families and intervention strategies

By interviewing seven expert volunteers we were able to identify key aspects to classify conversations. These “thematic families” describe overarching topics (semantic context) of each conversation. We identified six main thematic families:

- Relational themes: Conversations covering family, friends, partners, or lack thereof. These themes relate to issues of interpersonal relations and human bonds.
- Self-image themes: Conversations covering personal value, personal devaluation, over-demands, questions about self-identity. These themes relate to issues of personal worth and self-efficacy beliefs.
- Sexual diversity themes: Conversations covering gender identity, roles, sexual orientation, or sexual dissidence. These themes relate to sex and gender exploration.
- Performance themes: Conversations covering academic, financial, or other forms of perceived performance. These themes relate to success and failure dealing with external demands.
- Emotional crisis themes: Conversations covering emotional control, rage, liability, or emotional management. These themes relate to hardships of dealing with emotions of negative valence.
- Violence themes: Conversations covering sexual, physical, or emotional abuse, verbal or physical violence, abandonment, economic violence or infidelity or any other form of perceived violence. These themes relate to the multiple forms of interpersonal violence.

The classification into thematic families was part of the 1000 conversation tagging process. We used these results as inputs for logistic regression models, where the dependent variables were anxious and depressive symptomatology and suicidal behavior.

Additionally, we used these results to train a classifier for each thematic family, from which we derived a set of predictor terms. Table 1 shows the list of terms associated with each of the thematic families. The accuracy and precision metrics were estimated by comparing the dictionary-based classification with the expert’s tagging on the 1000 conversation database. Dictionary-based classification performance varies among different thematic families. The performance increases when the semantic families are less prone to contextual changes, i.e., if adding of new concepts contributes to saturating the family.

**Table 1:** List of terms and performance of thematic families.

Thematic family	Accuracy	Precision	Recall	Words
Self-image	0.73	0.66	0.58	Body, self-esteem, ugly, insecure, hate, negative, disgusted, graceful, attractive, rejection, eat, abnormal, fat, pretty, obesity, obese, self-image, beautify
Violence	0.86	0.77	0.52	Raped, rape, bullying, abuse, abused, violence, aggression, hitting, yelling, humiliate, harassment
Sexual diversity	0.93	0.82	0.74	Gay, homosexual, orientation, trans, bisexual, lesbian, closet, gender, homophobic, straight, transgender, binary, hermaphrodite, intersex, fluid, bisexual, demisexual, pansexual, non-gender
Relational	0.80	0.83	0.90	Friend, friends, single, alone, lonely, father, dad, family, relatives, parents, partner, boyfriend, girlfriend, discuss, finished, brothers, mother, mom, toxic, home
Emotional crisis	0.66	0.65	0.56	Crisis, cry, anxiety, panic, chest, anguish, breathing, sweating, trembling, tremble, pulse, fears
Performance	0.71	0.54	0.61	Career, study, salary, university, grades, test, insufficient, work, money, performance, effort, try

To use them as a control variable in our logistic models, we identified the theme of “Interventions" that describes the pragmatic responses that trained professional volunteers use to address different user scenarios. We identified seven main intervention options frequently used by volunteers:

- Exploration of the problem: Questions about the extent, origin, gravity, and expressions of mental health issues.
- Emotional containment: Regulation of emotions and calming of users.
- Identification of personal resources: Questions and assertions about personal networks, hobbies, interests and/or access to professional mental health services.
- Inducing reflection: Questions aimed at perspective-taking.
- Validation of personal experience: Generalization or normalization of user’s statements to validate them as being expected or normal.
- Psycho-education: Assertions aimed at explaining or conceptualizing the user’s subjective experience.
- Professional derivation: Suggestions about accessing other professional mental health services.

### Effect of the pandemic on the thematic content of the conversations

**Fig. 1:**
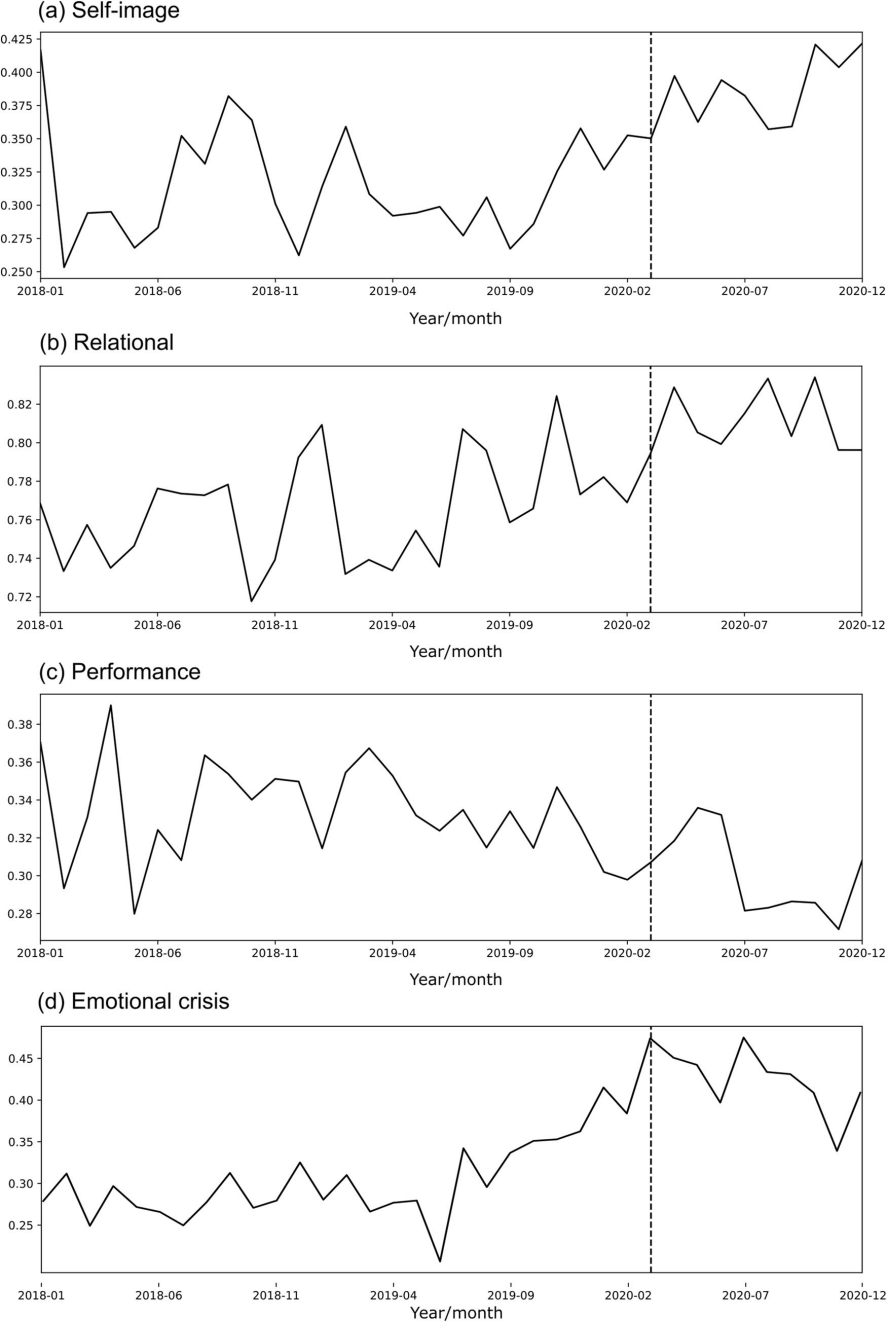
Theme prevalence, normalized by the total number of conversations in a month/year. The vertical dotted line indicates the starting of COVID-19 pandemic in Chile. **a** Self-image themes. **b** Relational themes. **c** Performance themes. **d** Emotional crisis themes.

Using the dictionary-based classification on the full dataset, we estimated the prevalence of each thematic family from 2018 to 2020 at the monthly level. The differences in prevalence before and during the pandemic were tested by setting up a first order autoregressive model, with a dummy variable indicating the pandemic months (from March 2020 onwards). A significant effect of the dummy at the 0.01 level was found in Self-image (*t* = 4.38, *p*-value = 1.2e−05), Relational (*t* = 4.52, *p*-value = 6.09e−06), Performance (*t* = −3.48, *p*-value = 4.9e−4) and Emotional crisis (*t* = 4.90, *p*-value = 8.83e−07), tested with a *t*-test. In all these cases, the residuals were not auto-correlated (tested with a Ljung-box test), which suggest an adequate model fit. A positive/negative value of *t* means an increase/decrease of theme prevalence during the pandemic. Figure 1 shows the evolution of these thematic families from 2018 to 2020. This period covers ten months of the pandemic in Chile, from March 2020 to December 2020.

Self-image: The pre-post-pandemic change may respond to the greater exposure to body and appearance issues. Looking at their own faces in virtual platforms all the time resembles a “mirror" on screen, allowing them to inspect their appearance simultaneously^47^. The zoom effect caused by videoconferencing systems raises body image concerns that are associated with self-focused attention and with increased concern about appearance and how to change it, due to time spent on video calls^48^. Looking at oneself during video chatting is associated with self-objectification and appearance comparison on face satisfaction and body satisfaction^47^. Also, exposure to weight-stigmatizing content on social media increased during the pandemic among adolescents^49^. Using hide self-view on videoconferencing systems and “touch up" features can increase bodily discomfort by having an ideal image on the screen^47,48^. Furthermore, daily routine disruptions, increased snacking and the lack of outdoor activities may raise weight and shape concerns^50^. In addition, the pandemic and social distance may have diminished social support and adaptative coping strategies, which may increase discomfort with the body^50^.

Relational: Family and social relations have suffered due to confinement, which could explain the change in this topic. An increase has also been reported in tensions between LGBT+ adolescents with their families^51^, and also among young adults with friends and partners^52^, because of the pandemic and confinement^53^.

Performance: The decrease in conversations about performance can be interpreted in different ways. On the one hand, previous studies have shown inconsistent results regarding the impact of COVID-19 on academic performance, with literature pointing to effects in both directions^54–57^. In this sense, these results should be carefully interpreted in terms of their implications in the context of academic performance. On the other hand, domestic monetary concerns may have been relaxed due to government money transfers and various government-authorized withdrawals from pension funds.

Emotional crisis: this topic showed the highest increase during the pandemic. Since the prevalence in thematic families is normalized by the total of monthly conversations, an increase in this topic means that, during the pandemic, there were more crisis-related calls to the helpline, compared with other types of calls. The latter includes conversations aiming for company, support and information-seeking calls.

Violence: As some studies reported an increase in domestic violence due to confinement^58–60^, we would expect a higher presence of this thematic family in the pandemic. However, our results show no difference pre-post pandemic. This suggests that the violence exerted on this segment of the population finds causes in contexts other than confinement. Also, mentions of violent situations are generally not complaints but rather arise as part of the narrative of other problems.

### Association of thematic families with mental health issues

Logistic regression models were fitted to the three mental health issues: suicidal behavior, anxious and depressive symptomatologies. The different intervention strategies and thematic families correspond to dichotomous predictive variables for these models. Table 2 shows the results of the logistic regression on each mental health issue. These models were trained with the 1000 conversation tagged dataset. As the date of the Pandemic outbreak could impact the dependent variable, we ran additional models with a dummy variable for the pandemic. Our primary results were not significantly affected (see Table 1 in Supplementary Information).

**Table 2:** Logistic regression results.

	Suicidal	Depressive	Anxious
Intercept	−0.693^***^	−1.354^***^	−2.269^***^
	(0.203)	(0.214)	(0.262)
Themes			
Self-image	0.405^***^	0.481^***^	−0.186
	(0.153)	(0.154)	(0.168)
Performance	−0.259	0.504^***^	0.060
	(0.159)	(0.159)	(0.170)
Sexual diversity	−0.554^***^	−0.492^**^	−0.012
	(0.202)	(0.198)	(0.212)
Emotional crisis	0.599^***^	0.802^***^	1.076^***^
	(0.148)	(0.150)	(0.164)
Relational	−0.350^**^	0.140	−0.190
	(0.170)	(0.171)	(0.187)
Violence	0.335^*^	0.422^**^	−0.170
	(0.172)	(0.179)	(0.189)
Strategies			
Emotional containment	0.529^***^	0.233	−0.125
	(0.185)	(0.178)	(0.203)
Professional derivation	0.290^*^	0.247	−0.130
	(0.163)	(0.171)	(0.179)
Psycho-education	0.231	−0.113	0.330^*^
	(0.179)	(0.185)	(0.186)
Exploration of the problem	−0.908^***^	−0.598^***^	0.253
	(0.209)	(0.213)	(0.252)
Identification of personal resources	0.503^***^	0.849^***^	0.336^**^
	(0.156)	(0.152)	(0.171)
Inducing reflection	0.050	−0.111	0.256
	(0.170)	(0.173)	(0.178)
Validation of personal experience	0.100	0.581^***^	0.730^***^
	(0.166)	(0.163)	(0.192)
Observations	1000	1000	1000
Pseudo *R*^2^	0.08938	0.1441	0.1027

The significance of each coefficient was tested using a *t*-test.

*p < 0.1; **p < 0.05; ***p < 0.01.

For suicidal behavior, the main positively associated themes are Emotional crisis and Self-image. While the former describes the crisis itself, the latter points to the motive. In other words, a self-image problem may generate—along with other factors—emotional management issues. The prevalence of both topics could respond to a “cause-effect" or “description-explanation" scheme. Although less significant, Violence is also a positive predictor of suicidal behavior. This may be due to a direct effect^61^, or to indirect effects on self-image^62^ or other suicide risk factors^63^. Both Relational and Sexual diversity themes are significant, but are negative predictors. In general, Sexual diversity has no positive effect in any model. While the *Safe Hour* program does not reject any call, this helpline is intended to serve as a psychological support for those LGBT+ people who need to talk to someone. Thus, many LGBT+ people (or even their relatives) use the helpline to ask for information and talk about sexual diversity.

Regarding the strategies, Emotional containment shows the highest positive association with suicidal behavior. This strategy corresponds to the immediate action in such a crisis, since the main objective is to stop the suicidal act. The Identification of personal resources also shows a positive and significant effect, with a slightly lower magnitude. This strategy may point to the crisis management itself - as in finding someone who can help in the crisis, for example, driving to the hospital - but also to the non-immediate causes. In this sense, personal resources can also correspond to activities and personal relations that contribute to personal welfare in daily life.

In the case of depressive symptomatology, several themes have a positive and significant effect. In order of importance, they are Emotional crisis, Performance, Self-image and Violence. This indicates that depressive tendencies are aggravated by multiple causes of a personal or relational nature. Again, Emotional crisis seems to describe the symptom itself. Regarding the strategies, the Identification of personal resources appears as the most important predictor, followed by Validation of personal experience. In this case, the identification of personal resources probably refers to the search of activities, interests and personal relations that help the user to better face the depressive episode. Validation of personal experience also plays a relevant role because greater validation is associated with decreased negative affects^64^.

From our sample of 1000 tagged conversations, suicidal behavior and depressive symptomatology are the only mental health conditions that show a positive and significant association. In this case, the correlation coefficient *ϕ* equals 0.29. This value can be tested for statistical significance with a *χ*^2^ test, and the resulting *p*-value is lower than 0.001. However, even when suicidal behavior and depressive symptomatologies show a significant association, the strategy scheme in each case is slightly different. The element that separates them is the preponderance of containment in suicidal behavior and validation as a relevant element in depressive behavior.

Finally, only the theme of Emotional Crisis has a significant and positive effect on anxious symptomatology. This suggests that people with this symptomatology usually use the platform in a crisis context, probably as a last resource. This is consistent with the fact that clinical anxiety provokes cognitive and affective hyperreactivity, increases the perceived intensity of emotional experiences, perceived difficulties in managing negative mood states and experiences of overwhelming worry^65^. In this sense, it makes sense that users’ discourse is centered around emotional management themes that may be masking other underlying issues.

Regarding strategies, Validation of personal experience is the most important predictor, followed by Identification of personal resources. Psycho-education also appears, but with low significance, although its magnitude is similar to Identification of personal resources. Since Psycho-education and Inducing reflection strategies have an *ϕ* correlation coefficient of 0.39 (the highest association among symptomatologies, themes and strategies), we ran the model without the Inducing reflection strategy, in which case Psycho-education becomes significant at the 5% level, and takes second place after Validation of personal experience.

## Discussion

This article set out to show the efficacy of alternative means to conduct population studies on mental health. Considering the high cost of survey-based approaches, the black-boxing produced by unsupervised machine learning, and the de-contextualization produced by off-the-shelve dictionaries, our manuscript argues for the use of interdisciplinary approaches driven by qualitative understanding.

We employed qualitative interviews to identify the psychosocial stressors present in the helpline conversations. We were able to map the topics of conversation (thematic families) that operate as the source of distress beyond the type of distress (depressive and anxious symptomatologies or suicidal behavior). These included relational, self-image, sexual diversity, emotional crisis, and violence themes. We also identified the strategies used by volunteers to address the concerns of users. These ranged from helping users explore their problems to providing psychological education. Future studies may use these sorts of categories in combination with other frequently assessed variables in text-based psychological assessment, such as personality traits^66,67^. Incorporating more variables with strong empirical support is especially critical if an automatic analysis is used at the individual level and not only for large cohorts. We then operationalized our qualitative findings using both expert human taggers and automatic computational methods. This enabled us to describe how topics of conversations changed during the COVID-19 crisis, and how mental health symptomatologies relate to conversation themes.

We observed how the thematic composition of conversations changed before and during the COVID-19 crisis. Relational and self-image issues were more dominant during COVID-19. This first insight possibly reflects the increase of home interpersonal relationships and the decrease of in-person friend interactions. The interpretation of the increase of self-image issues is more complex but perhaps points to the manifestation of personal life through social media, which raises questions of self-worth, particularly among adolescents. The decrease of performance issues relates to the inconsistent findings of the effects of COVID-19 in educational settings, and the provision of economic aid by the national government. Overall, these findings show that although the dictionaries are contextually bounded, they enable discussions with previous evidence on a global scale.

Gathering mental health conversations from chats means that the nature of the data is dialogical instead of monological. That means that the texts’ characteristics are modified both by users and volunteers. Controlling by the volunteers’ interventions, we associated how thematic families link to mental health symptoms. In all three depressive, anxious, and suicidal cases, emotional crisis was a major topic of conversation. For depressive and suicidal symptoms, self-image themes were also relevant. Performance and violence themes were also significantly related to depressive symptoms. These findings highlight the need to contextualize population mental health issues within their semantic content; in other words, understanding the sources and context of distress, rather than only symptomatologies. For instance, depressive symptoms are associated with more themes, possibly reflecting that a person is more likely affected by different sources of life’s challenges. On the other hand, anxiety symptoms are related primarily to emotional management, indicating that a person is focusing on the immediate discomfort and consequences of the mental health crisis.

However, this study does have some limitations. Although the use of chat records proved to yield results consistent with previous evidence, population studies should include multiple data sources to have the validity required for policy decision-making. The purpose of this study was not to produce such results but to showcase how that sort of data could be assessed. On the other hand, human taggers validated our results, which led to classification bias. We sought to mitigate that error by not only selecting taggers with domain expertise, but also by testing them beforehand in order to assess their reliability. Nonetheless, many of the findings are dependent on their personal judgment, which should be considered when interpreting our results. As a population study, our methodological approach is meant for assessing large cohorts and not individual people. More variables and larger data sources would be necessary for making interpretations at the individual level.

Other limitations arise from the methods we used. The incorporation of individual-level control variables may contribute towards understanding the associations between mental health issues and different thematic families. Regarding the prevalence of thematic families, our dataset does not cover a full pandemic year, and therefore it is not possible to incorporate seasonal effects in the analysis. Finally, the texts come from a highly informal context, in which spelling errors are very common, affecting the performance of dictionary-based methods.

### Supplementary information

Editorial Policy Checklist

Reporting Summary

Supplementary Information

## Data Availability

The data that support the findings of this study are available from Fundación Todo Mejora but restrictions apply to the availability of said data, which were used under license for the current study, and so are not publicly available. Data are however available from the authors upon reasonable request and with the permission of Fundación Todo Mejora.

## Code availability

The computer codes that support the findings of this study are publicly available at: https://github.com/GobLab-UAI/Proyecto-COVID1006-UAI-Fundacion-Todo-Mejora.
