# Supplementary material for: Natural language processing analysis of the psychosocial stressors of mental health disorders during the pandemic
Source: Npj Ment Health Res. 2023 Oct 5;2:17. doi: 10.1038/s44184-023-00039-6 (PMC10955824; doi:10.1038/s44184-023-00039-6)
Supplement: Supplementary file 2 — Reporting Summary [file 44184_2023_39_MOESM2_ESM.pdf]

nature portfolio

Corresponding author(s): María-Paz Raveau

Last updated by author(s): May 29, 2023

Reporting Summary

Nature Portfolio wishes to improve the reproducibility of the work that we publish. This form provides structure for consistency and transparency in reporting. For further information on Nature Portfolio policies, see our Editorial Policies and the Editorial Policy Checklist.

Statistics

For all statistical analyses, confirm that the following items are present in the figure legend, table legend, main text, or Methods section.

n/a	Confirmed	
☐	☒	The exact sample size (*n*) for each experimental group/condition, given as a discrete number and unit of measurement
☒	☐	A statement on whether measurements were taken from distinct samples or whether the same sample was measured repeatedly
☐	☒	The statistical test(s) used AND whether they are one- or two-sided<br>*Only common tests should be described solely by name; describe more complex techniques in the Methods section.*
☐	☒	A description of all covariates tested
☒	☐	A description of any assumptions or corrections, such as tests of normality and adjustment for multiple comparisons
☐	☒	A full description of the statistical parameters including central tendency (e.g. means) or other basic estimates (e.g. regression coefficient) AND variation (e.g. standard deviation) or associated estimates of uncertainty (e.g. confidence intervals)
☐	☒	For null hypothesis testing, the test statistic (e.g. *F*, *t*, *r*) with confidence intervals, effect sizes, degrees of freedom and *P* value noted<br>*Give P values as exact values whenever suitable.*
☒	☐	For Bayesian analysis, information on the choice of priors and Markov chain Monte Carlo settings
☒	☐	For hierarchical and complex designs, identification of the appropriate level for tests and full reporting of outcomes
☒	☐	Estimates of effect sizes (e.g. Cohen's *d*, Pearson's *r*), indicating how they were calculated

Our web collection on statistics for biologists contains articles on many of the points above.

Software and code

Policy information about availability of computer code

Data collection	No software was used to collect the data. This study uses an existing dataset, the helpline chat data from the Safe Hour program of Fundación Todo Mejora (It Gets Better Project) in Chile.
Data analysis	The computer codes that support the findings of this study are publicly available at: https://github.com/GobLab-UAI/Proyecto-COVID1006-UAI-Fundacion-Todo-Mejora.

For manuscripts utilizing custom algorithms or software that are central to the research but not yet described in published literature, software must be made available to editors and reviewers. We strongly encourage code deposition in a community repository (e.g. GitHub). See the Nature Portfolio guidelines for submitting code & software for further information.

Data

Policy information about availability of data

All manuscripts must include a data availability statement. This statement should provide the following information, where applicable:

- Accession codes, unique identifiers, or web links for publicly available datasets
- A description of any restrictions on data availability
- For clinical datasets or third party data, please ensure that the statement adheres to our policy

The data that support the findings of this study are available from Fundación Todo Mejora but restrictions apply to the availability of these data, which were used

under license for the current study, and so are not publicly available. Data are however available from the authors upon reasonable request and with permission of Fundación Todo Mejora.

Research involving human participants, their data, or biological material

Policy information about studies with human participants or human data. See also policy information about sex, gender (identity/presentation), and sexual orientation and race, ethnicity and racism.

Reporting on sex and gender	This information has not been collected.
Reporting on race, ethnicity, or other socially relevant groupings	This information has not been collected.
Population characteristics	Participants can freely decide to share personal information when reaching out to the helpline. 4643 of 7640 participants revealed their age. Out of these participants, the average age was 18.89 years old (5.17 years Standard Deviation).
Recruitment	The dataset comes from a helpline chat, so participants were self-selected (each participant decided whether to use or not the helpline).
Ethics oversight	Comité Ético de Investigación, Universidad Adolfo Ibáñez, number N°02/2021

Note that full information on the approval of the study protocol must also be provided in the manuscript.

Field-specific reporting

Please select the one below that is the best fit for your research. If you are not sure, read the appropriate sections before making your selection.

☐ Life sciences ☒ Behavioural & social sciences ☐ Ecological, evolutionary & environmental sciences

For a reference copy of the document with all sections, see nature.com/documents/nr-reporting-summary-flat.pdf

Behavioural & social sciences study design

All studies must disclose on these points even when the disclosure is negative.

Study description	Mixed-methods sequential exploratory approach, with a qualitative stage (semi-structured in-depth interviews) and quantitative analysis.
Research sample	This study uses an existing dataset, the helpline chat data from the Safe Hour program of Fundación Todo Mejora (It Gets Better Project) in Chile.
Sampling strategy	The dataset comes from a helpline chat, so participants were self-selected (each participant decided whether to use or not the helpline).
Data collection	This study uses an existing dataset, the helpline chat data from the Safe Hour program of Fundación Todo Mejora (It Gets Better Project) in Chile.
Timing	The dataset used for this study covers a period of 2018 to 2020.
Data exclusions	The dataset comes from a helpline chat, so participants were self-selected (each participant decided whether to use or not the helpline).
Non-participation	The dataset comes from a helpline chat, so participants were self-selected (each participant decided whether to use or not the helpline).
Randomization	The dataset comes from a helpline chat, so participants were self-selected (each participant decided whether to use or not the helpline).

Reporting for specific materials, systems and methods

We require information from authors about some types of materials, experimental systems and methods used in many studies. Here, indicate whether each material, system or method listed is relevant to your study. If you are not sure if a list item applies to your research, read the appropriate section before selecting a response.

Materials & experimental systems

n/a	Involved in the study	
☒	☐	Antibodies
☒	☐	Eukaryotic cell lines
☒	☐	Palaeontology and archaeology
☒	☐	Animals and other organisms
☒	☐	Clinical data
☒	☐	Dual use research of concern
☒	☐	Plants

Methods

n/a	Involved in the study	
☒	☐	ChIP-seq
☒	☐	Flow cytometry
☒	☐	MRI-based neuroimaging
