# Supplementary material for: Natural language processing analysis of the psychosocial stressors of mental health disorders during the pandemic
Source: Npj Ment Health Res. 2023 Oct 5;2:17. doi: 10.1038/s44184-023-00039-6 (PMC10955824; doi:10.1038/s44184-023-00039-6)
Supplement: Supplementary file 3 — Supplementary Information [file 44184_2023_39_MOESM3_ESM.pdf]

Supplementary information

Supplementary Table 1: Logistic regression results, with pandemic dummy. Conversations carried out from march 2020 onwards were identified as pandemic. Note: *p<0.1; **p<0.05; ***p<0.01

	Suicidal	Depressive	Anxious
Intercept	-0.624***	-1.333***	-2.296***
	(0.205)	(0.216)	(0.263)
Themes			
Self-image	0.443***	0.491***	-0.199
	(0.155)	(0.154)	(0.169)
Performance	-0.281*	0.499***	0.068
	(0.160)	(0.159)	(0.170)
Sexual diversity	-0.535***	-0.485**	-0.021
	(0.203)	(0.198)	(0.212)
Emotional crisis	0.630***	0.813***	1.063***
	(0.149)	(0.151)	(0.165)
Relational	-0.359**	0.139	-0.190
	(0.171)	(0.171)	(0.187)
Violence	0.327*	0.418**	-0.164
	(0.173)	(0.179)	(0.189)
Strategies			
Emotional containment	0.569***	0.242	-0.138
	(0.187)	(0.178)	(0.204)
Professional derivation	0.230	0.229	-0.103
	(0.165)	(0.172)	(0.181)
Psycho-education	0.265	-0.102	0.318*
	(0.180)	(0.186)	(0.187)
Exploration of the problem	-0.923***	-0.601***	0.251
	(0.210)	(0.213)	(0.252)
Identification of personal resources	0.493***	0.845***	0.343**
	(0.156)	(0.152)	(0.171)
Inducing reflection	0.048	-0.112	0.259
	(0.171)	(0.173)	(0.178)
Validation of personal experience	0.128	0.590***	0.719***
	(0.167)	(0.164)	(0.192)
Pandemic	-0.436***	-0.132	0.171
	(0.166)	(0.166)	(0.172)
Observations	1,000	1,000	1,000
Pseudo R^2	0.09465	0.1446	0.1035
